# Correspondence

**Published:** 1878-10

**Authors:** J. G. Templeton


### Correspondence.

### CORRESPONDENCE.

EDITOR REGISTER:—I write for the purpose of saying a few words in behalf of an automatic plugger which I have had in daily use for nearly two years and which is called the "King Dental Malleter," invented by Dr. Courtland King, of this city. And in my humble opinion it is indeed *the* king of automatics. Always ready for use with a force so easily and so completely controlled by the will of the operator through a forward motion of the index finger turning and controlling the light, wooden stem with the thumb and second finger; all of which is done so easily that he soon forgets that he is exerting any effort to produce a blow so effective in welding or condensing gold, which it does to my satisfaction better than any other mallet that I have had or seen in use.

The ease with which it is controlled, together with the readiness with which it responds with its condensing force at the precise time and place desired, inspires such a confidence that we can pack gold against the frailest side of a cavity without the least fear of fracturing that wall. Another great advantage this mallet possesses is that the blow is obtained without pressure against the gold, consequently the stroke does not inflict pain like one following pressure the reason of which is obvious, as by any force brought to bear against a tooth compresses the peridential membrane against the opposite side of the socket, and a succession of blows under such a condition is liable in many cases to be productive of periostitis. Hence we conclude that the advantage is largely in favor of the instrument that will do its work without any force being brought to bear against the tooth to produce the condensing stroke. Such are the qualities in favor of the "King Dental Malleter," its stroke producing no movement in the tooth beyond what is normal, from which it recovers like a cushion and without pain to the patient.

From the experience I have had with this instrument I have no hesitation in saying that I consider it one of "the trio of indispensables," viz.: "King Dental Malleter," "Rubber Dam," "Burring Engine." J. G. TEMPLETON.

---

